# The effects of socioeconomic and geographic factors on chronic phase long-term survival after stroke in South Korea

**DOI:** 10.1038/s41598-022-08025-2

**Published:** 2022-03-14

**Authors:** Dougho Park, Su Yun Lee, Eunhwan Jeong, Daeyoung Hong, Mun-Chul Kim, Jun Hwa Choi, Eun Kyong Shin, Kang Ju Son, Hyoung Seop Kim

**Affiliations:** 1 Department of Rehabilitation Medicine, Brain and Vascular Center, Pohang Stroke and Spine Hospital, Pohang, Republic of Korea; 2 Department of Neurology, Brain and Vascular Center, Pohang Stroke and Spine Hospital, Pohang, Republic of Korea; 3 Department of Neurosurgery, Brain and Vascular Center, Pohang Stroke and Spine Hospital, Pohang, Republic of Korea; 4 Department of Quality Improvement, Pohang Stroke and Spine Hospital, Pohang, Republic of Korea; 5 Department of Sociology, Korea University, Seoul, Republic of Korea; 6 Department of Research and Analysis, National Health Insurance Service Ilsan Hospital, Goyang, Republic of Korea; 7 Department of Biostatistics and Computing, Yonsei University Graduate School, Seoul, Republic of Korea; 8 Department of Physical Medicine and Rehabilitation, National Health Insurance Service Ilsan Hospital, Goyang, Republic of Korea

**Keywords:** Epidemiology, Stroke

## Abstract

The stroke incidence has increased rapidly in South Korea, calling for a national-wide system for long-term stroke management. We investigated the effects of socioeconomic status (SES) and geographic factors on chronic phase survival after stroke. We retrospectively enrolled 6994 patients who experienced a stroke event in 2009 from the Korean National Health Insurance database. We followed them up from 24 to 120 months after stroke onset. The endpoint was all-cause mortality. We defined SES using a medical-aid group and four groups divided by health insurance premium quartiles. Geographic factors were defined using Model 1 (capital, metropolitan, city, and county) and Model 2 (with or without university hospitals). The higher the insurance premium, the higher the survival rate tended to be (*P* < 0.001). The patient survival rate was highest in the capital city and lowest at the county level (*P* < 0.001). Regions with a university hospital(s) showed a higher survival rate (*P* = 0.006). Cox regression revealed that the medical-aid group was identified as an independent risk factor for chronic phase mortality. Further, NHIP level had a more significant effect than geographic factors on chronic stroke mortality. From these results, long-term nationwide efforts to reduce inter-regional as well as SES discrepancies affecting stroke management are needed.

## Introduction

Along with population aging, the prevalence of stroke is on the rise. The American Heart Association represented the global prevalence of cerebrovascular disease (CVD) was 101.5 million people, and CVD attributed to 6.6 million deaths in 2019^1^. According to 2018 Stroke Statistics in Korea, one in 40 adults experiences a stroke, with 232 individuals per 100,000 people undergoing stroke every year^2^. According to statistics on the causes of deaths released by the Korean National Statistical Office, 42 deaths per 100,000 people were due to CVD as of 2019 and is ranked fourth as the cause of death after malignant neoplasms, cardiovascular diseases, and pneumonia^3^. This aging population and the accompanying increase in CVD prevalence generate an increasingly onerous socioeconomic burden^4^. Therefore, establishing a national-wide system to manage CVD effectively has become more imperative.

Studies across countries have examined long-term survival and related risk factors in stroke survivors such as age, male sex, comorbidities, and disabilities; however, most studies included death in both the acute and chronic phases^5–7^. This means that their survival analysis could be considerably influenced by the high mortality rate occurring in the acute phase. Few studies have exclusively observed patients surviving longer than a certain period after stroke onset. Therefore, large-scale analyses focusing on long-term stroke survivors are warranted.

Several studies have reported that geographic and socioeconomic factors are risk factors related to death after stroke^8–11^. Because immediate treatment following a stroke event is critical, access to a nearby medical institution on stroke onset is important for successful management^12^. Currently, tertiary and general hospitals are generally concentrated in large urban areas in the Korean healthcare system^13^. Therefore, it can be assumed that residential location is likely to reflect overall medical accessibility and the quality of medical services provided to patients. Additionally, South Korea has implemented a national health insurance service (NHIS) in the form of social insurance funded through compulsory contributions from all citizens, divided into medical-aid and NHIS-covered categories^14^. Medical-aid is a public assistance program run by the government that guarantees medical assistance for low-income citizens. Among NHIS beneficiaries, the payment type is divided into two types: self-employed and employed^15,16^. Premium levels are generally determined by each household's income or assets. Specifically, employed insurance is only based on earned income, while self-employed insurance is based on unearned income, real estate, and car ownership^15^.

Based on these assumptions, this study attempted to analyze the effects of national health insurance premium (NHIP) level and residential area (serving as proxy indicators of socioeconomic and geographic factors, respectively) on chronic phase long-term survival in patients with stroke. In addition, we also aimed to identify independent risk factors that affect chronic phase mortality after stroke under the Korean NHIS.

## Methods

### Data source and patients

We conducted a retrospective longitudinal study using a population-based cohort dataset obtained from the NHIS (Research management number: NHIS-2020-1-160). This study design was reviewed and approved by the Institutional Review Board of National Health Insurance Service Ilsan Hospital (Approval number: NHIS 2021-1-577). Informed consent was waived due to the retrospective nature of this study by the Institutional Review Board of National Health Insurance Service Ilsan Hospital. This study was conducted in compliance with the Declaration of Helsinki.

**Figure 1:**
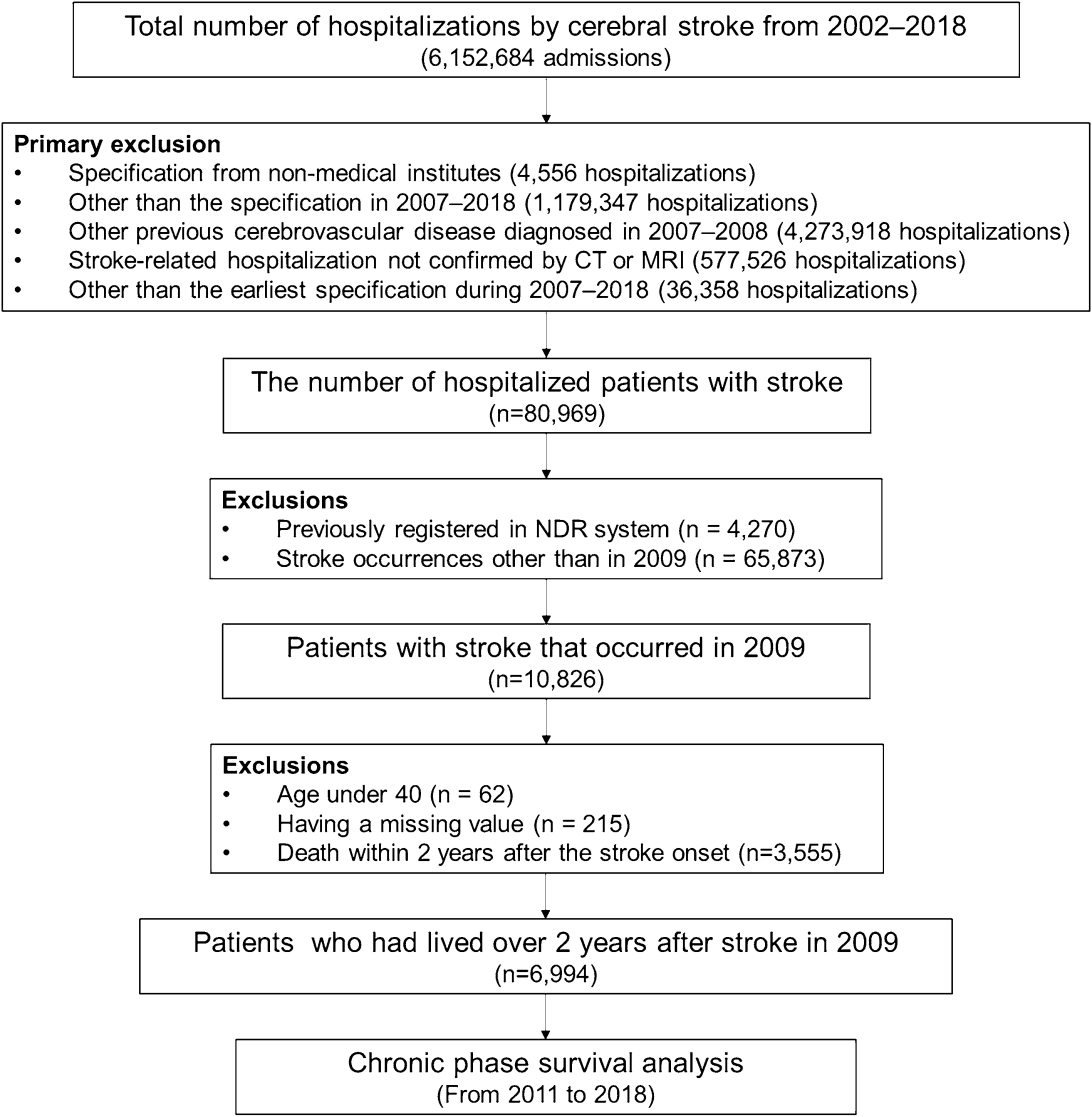
The flow-chart of this study.

Figure 1 shows the flowchart of the study. The initial sample consisted of 6,152,684 specifications for stroke hospitalization. Using International Classification of Diseases (ICD)-10 codes, we defined stroke-related diagnosis as I60–I64. Primary exclusions were as follows: (1) specifications from non-medical institutes (from other than tertiary hospitals, general hospitals, hospitals, rehabilitation hospitals, convalescent hospitals, private clinics, and public health institutions), (2) admissions outside the years 2007–2018, (3) previous cerebrovascular diseases during 2007–2008 (ICD-10 codes: G46 and I60–69), (4) stroke diagnosis not confirmed by computed tomography or magnetic resonance imaging, and (5) not the earliest hospitalization in the sampling period. After primary exclusions, 80,969 patients were selected. We excluded patients already registered as disabled in the National Disability Registration (NDR) system due to brain disorders and patients who had experienced strokes during years other than 2009. Among the 10,826 patients with stroke that had occurred in 2009, we additionally excluded patients under 40 years of age, with missing values, and who had died within two years after the stroke event. Following these exclusions, 6994 patients were included in the final cohort for the study. They were followed up for ten years after stroke onset and analyzed for this study.

### Study variables

Under the Korean NHIS, a patient’s NHIP level can be a reliable surrogate for measuring their economic status. Therefore, to define socioeconomic status, we used NHIP levels to establish four different quartile groups (1st–4th quartile groups), along with a medical-aid group. We selected residential areas as the geographic factor of interest and then set up two models. For Model 1, we designated the following four categories: capital (Seoul), metropolitan, city, and county. For Model 2, we designated two categories, namely, administrative districts with at least one or more university hospitals (AD with UH) and administrative districts without a university hospital (AD without UH). The distribution of AD with UH in South Korea is shown in Supplementary Table 1.

We specified gender, age, and comorbidities as baseline characteristics. We focused on patients aged ≥ 40 and distinguished age groups by decade. In terms of comorbidities, we selected hypertension, diabetes, dyslipidemia, coronary artery disease (CAD), atrial flutter/fibrillation (AF), and chronic kidney disease (CKD). Supplementary Table 2 shows the ICD-10 codes for these comorbidities.

In terms of stroke-related variables, strokes were categorized into the following subtypes: subarachnoid hemorrhage (I60), intracranial hemorrhage (I61 and I62), cerebral infarction (I63), and unspecified subtype (I64). We defined the stroke onset as the date of specification's commencement that satisfies the following conditions: (1) admission with stroke-related ICD-10 codes and (2) claim codes of brain MRI or CT for disease confirmation. The NDR grade was used to assess the degree of disability after stroke. All patients with stroke can apply for registration in the NDR system from six months after stroke onset^17^. The government classifies patients disabled by stroke into six grades depending on their level of daily activity, mainly based on the Modified Barthel Index, wherein the lower the grade, the more severe the degree of disability. If the criterion for disability is not met, such a patient is classified as ungraded. Supplementary Table 3 shows NDR grading system classifications according to the Modified Barthel Index.

### Statistical analysis

Categorical variables were expressed as frequency and proportion. A Chi-squared test was performed to determine the difference in frequency between the groups in each variable. The dependent variable was set as all-cause mortality within 24 to 120 months from stroke onset. Time to death was identified and used for survival analysis. To determine cumulative chronic phase survival, we performed a Kaplan–Meier survival analysis with log-rank tests according to NHIP levels and residential areas. Cox regression analyses were used to identify independent risk factors related to chronic phase mortality after stroke. Results were considered statistically significant with *P*-values of < 0.05. All statistical analyses were performed using SAS version 9.4 (SAS Institute, Inc., Cary, NC, USA).

## Results

### Baseline characteristics according to NHIP levels

Table 1 shows the characteristics of the patients according to NHIP levels. The monthly NHIP amount in relation to each premium quartile level in 2018 is presented in Supplementary Table 4.

**Table 1:** Baseline characteristics according to the NHIP levels.

	National health insurance premium levels					*P*-value
	Medical-aid (n = 853)	1st quartile (n = 1016)	2nd quartile (n = 990)	3rd quartile (n = 1452)	4th quartile (n = 2683)	
**Age groups, n (%)**						< 0.001
40–49	39 (4.57)	36 (3.54)	50 (5.05)	40 (2.75)	53 (1.98)	
50–59	87 (10.20)	123 (12.11)	136 (13.74)	174 (11.98)	182 (6.78)	
60–69	132 (15.47)	290 (28.54)	254 (25.66)	431 (29.68)	505 (18.82)	
70–79	348 (40.80)	333 (32.78)	346 (34.95)	557 (38.36)	1277 (47.60)	
≥ 80	247 (28.96)	234 (23.03)	204 (20.61)	250 (17.22)	666 (24.82)	
Male, n (%)	281 (32.94)	413 (40.65)	430 (43.43)	647 (44.56)	1101 (41.04)	< 0.001
**Stroke subtypes, n (%)**						0.004
SAH	16 (1.88)	19 (1.87)	47 (4.75)	56 (3.86)	81 (3.02)	
ICH	81 (9.50)	130 (12.80)	120 (12.12)	185 (12.74)	281 (10.47)	
Cerebral infraction	725 (84.99)	842 (82.87)	787 (79.49)	1167 (80.37)	2234 (83.27)	
Unspecified	31 (3.63)	25 (2.46)	36 (3.64)	44 (3.03)	87 (3.24)	
**Residential areas, n (%)**						< 0.001
Capital	87 (10.20)	182 (17.91)	139 (14.04)	218 (15.01)	472 (17.59)	
Metropolitan	177 (20.75)	206 (20.28)	179 (18.08)	276 (19.01)	534 (19.90)	
City	373 (43.73)	439 (43.21)	466 (47.07)	663 (45.66)	1238 (46.14)	
County	216 (25.32)	189 (18.6)	206 (20.81)	295 (20.32)	439 (16.36)	
AD with UH	435 (51.0)	590 (58.07)	551 (55.61)	816 (56.2)	1619 (60.34)	< 0.001
Hypertension, n (%)	653 (76.55)	815 (80.22)	768 (77.58)	1147 (78.99)	2160 (80.51)	0.070
Diabetes, n (%)	356 (41.74)	458 (45.08)	420 (42.42)	625 (43.04)	1223 (45.58)	0.164
Dyslipidemia, n (%)	331 (38.80)	436 (42.91)	398 (40.20)	623 (42.91)	1302 (48.53)	< 0.001
CAD, n (%)	83 (9.73)	78 (7.68)	80 (8.08)	144 (9.92)	300 (11.18)	0.006
AF, n (%)	38 (4.45)	64 (6.30)	66 (6.67)	117 (8.06)	245 (9.13)	< 0.001
CKD, n (%)	24 (2.81)	22 (2.17)	25 (2.53)	45 (3.10)	73 (2.72)	0.709
**Disability grades, n (%)**						0.009
Ungraded	798 (93.55)	878 (86.42)	875 (88.38)	1271 (87.53)	2374 (88.48)	
Grade 6	2 (0.23)	8 (0.79)	4 (0.40)	8 (0.55)	13 (0.48)	
Grade 5	5 (0.59)	5 (0.49)	7 (0.71)	15 (1.03)	18 (0.67)	
Grade 4	7 (0.82)	19 (1.87)	18 (1.82)	33 (2.27)	43 (1.60)	
Grade 3	11 (1.29)	29 (2.85)	16 (1.62)	42 (2.89)	54 (2.01)	
Grade 2	11 (1.29)	30 (2.95)	27 (2.73)	29 (2.00)	61 (2.27)	
Grade 1	19 (2.23)	47 (4.63)	43 (4.34)	54 (3.72)	120 (4.47)	

*SAH* subarachnoid hemorrhage, *ICH* intracranial hemorrhage, *AD* administrative districts, *UH* university hospital, *CAD* coronary artery disease, *AF* atrial flutter/fibrillation, *CKD* chronic kidney disease.

The mean age of patients was 71.67 ± 10.22 years old. The ratio of patients over 80 was highest in the medical-aid group (28.96%), and over 70 was highest (72.42%) in the 4th quartile group, which had the highest NHIP level when compared to the other groups (*P* < 0.001). The proportion of males was generally low, particularly in the medical-aid group (32.94%, *P* < 0.001). The ratios of patients with dyslipidemia (48.53%), CAD (11.18%), and AF (9.13%) were highest in the 4th quartile group (*P* < 0.001, *P* = 0.006, and *P* < 0.001, respectively). The distribution of hypertension, diabetes, and CKD did not show any significant difference between the groups.

In terms of regional distribution, the medical-aid group had the lowest percentage of patients living in the capital and metropolitan areas (30.95%). The 1st quartile group had the highest ratio of patients living in the capital and metropolitan areas (38.19%), followed by the 4th quartile group (37.49%) (*P* < 0.001). The medical-aid group had the lowest (51.0%), and the 4th quartile group had the highest (60.34%, *P* < 0.001) percentage of patients living in AD with UH.

Regarding the stroke subtype, the ratios of ischemic stroke were high in all groups. The hemorrhagic stroke ratio was highest in the 2nd quartile group (16.87%, *P* = 0.004). The ungraded ratio as determined in the NDR system was highest in the medical-aid group (93.55%). Regarding percentages of patients with relatively severe disabilities (that is, with NDR grades 1, 2, and 3), the 1st quartile group showed the highest percentage of such patients (10.43%), followed by the 4th quartile group (8.75%, *P* = 0.009).

### Baseline characteristics according to residential areas

Patient characteristics according to residential areas are summarized in Table 2. The distribution of populations in 2018 in Model 1 according to our definition of residential areas is presented in Supplementary Table 5.

**Table 2:** Baseline characteristics according to the residential areas.

	Residential areas							
	Capital (n = 1098)	Metropolitan (n = 1372)	City (n = 3179)	County (n = 1345)	*P*-value	AD with UH (n = 4011)	AD without UH (n = 2983)	*P*-value
**Age groups, n (%)**					< 0.001			< 0.001
40–49	37 (3.37)	43 (3.13)	114 (3.59)	24 (1.78)		134 (3.34)	84 (2.82)	
50–59	130 (11.84)	172 (12.54)	313 (9.85)	87 (6.47)		459 (11.44)	243 (8.15)	
60–69	271 (24.68)	336 (24.42)	731 (22.99)	275 (20.45)		981 (24.46)	631 (21.15)	
70–79	413 (37.61)	523 (38.12)	1299 (40.86)	626 (46.54)		1,540 (38.39)	1,321 (44.28)	
≥ 80	247 (22.50)	299 (21.79)	722 (22.71)	333 (24.76)		897 (22.36)	704 (23.60)	
Male, n (%)	480 (43.72)	595 (43.73)	1298 (40.83)	499 (37.10)	0.002	1,711 (42.66)	1,161 (38.92)	0.002
**Stroke subtypes, n (%)**					< 0.001			0.003
SAH	49 (4.46)	49 (3.57)	80 (2.52)	41 (3.05)		140 (3.49)	79 (2.65)	
ICH	149 (13.57)	167 (12.17)	357 (11.23)	124 (9.22)		494 (12.32)	303 (10.16)	
Cerebral infraction	852 (77.60)	1132 (82.51)	2613 (82.76)	1140 (84.76)		3,259 (81.25)	2,496 (83.67)	
Unspecified	48 (4.37)	24 (1.75)	111 (3.49)	40 (2.97)		118 (2.94)	105 (3.52)	
**NHIP levels, n (%)**					< 0.001			< 0.001
Medical-aid	87 (7.92)	177 (12.90)	373 (11.73)	216 (16.06)		435 (10.85)	418 (14.01)	
1st quartile	182 (16.58)	206 (15.01)	439 (13.81)	189 (14.05)		590 (14.71)	426 (14.28)	
2nd quartile	139 (12.66)	179 (13.05)	466 (14.66)	206 (15.32)		551 (13.74)	439 (14.72)	
3rd quartile	218 (19.85)	276 (20.12)	663 (20.86)	295 (21.93)		816 (20.34)	636 (21.32)	
4th quartile	472 (42.99)	534 (38.92)	1238 (38.94)	439 (32.64)		1,619 (40.36)	1,064 (35.67)	
Hypertension, n (%)	892 (81.24)	1088 (79.30)	2544 (80.03)	1019 (75.76)	0.003	3,220 (80.28)	2,323 (77.87)	0.014
Diabetes, n (%)	523 (47.63)	611 (44.53)	1417 (44.57)	531 (39.48)	< 0.001	1,850 (46.12)	1,232 (41.30)	< 0.001
Dyslipidemia, n (%)	590 (53.73)	601 (43.80)	1399 (44.01)	500 (37.17)	< 0.001	1,902 (47.42)	1,188 (39.83)	< 0.001
CAD, n (%)	129 (11.75)	137 (9.99)	288 (9.06)	131 (9.74)	0.080	411 (10.25)	274 (9.19)	0.140
AF, n (%)	83 (7.56)	115 (8.38)	246 (7.74)	86 (6.39)	0.254	320 (7.98)	210 (7.04)	0.143
CKD, n (%)	38 (3.46)	43 (3.13)	89 (2.80)	19 (1.41)	0.007	131 (3.27)	58 (1.94)	< 0.001
**Disability grades, n (%)**					0.104			0.107
Ungraded	959 (87.34)	1197 (87.24)	2818 (88.64)	1222 (90.86)		3,521 (87.78)	2,675 (89.67)	
Grade 6	9 (0.82)	9 (0.66)	12 (0.38)	5 (0.37)		24 (0.60)	11 (0.37)	
Grade 5	11 (1.00)	11 (0.80)	20 (0.63)	8 (0.59)		30 (0.75)	20 (0.67)	
Grade 4	24 (2.19)	24 (1.75)	51 (1.60)	21 (1.56)		67 (1.67)	53 (1.78)	
Grade 3	18 (1.64)	43 (3.13)	69 (2.17)	22 (1.64)		96 (2.39)	56 (1.88)	
Grade 2	25 (2.28)	28 (2.04)	76 (2.39)	29 (2.16)		91 (2.27)	67 (2.25)	
Grade 1	52 (4.74)	60 (4.37)	133 (4.18)	38 (2.83)		182 (4.54)	101 (3.39)	

*AD* administrative districts, *UH* university hospital, *SAH* subarachnoid hemorrhage, *ICH* intracranial hemorrhage, *NHIP* national health insurance premium, *CAD* coronary artery disease, *AF* atrial flutter/fibrillation, *CKD* chronic kidney disease.

The percentage of those over 70 in the county resident group was 71.3%, relatively high compared to the other groups (*P* < 0.001). The smaller the residential area, the lower the ratio of males tended to be (*P* = 0.002). In terms of hypertension, diabetes, dyslipidemia, and CKD, the capital resident group showed the highest ratio of patients while the county resident group showed the lowest (*P* = 0.003, *P* < 0.001, *P* < 0.001, and *P* = 0.007, respectively). There was no significant difference between the groups in terms of CAD and AF. Furthermore, the larger the residential area, the higher the NHIP level of residents (*P* < 0.001).

The ratio of hemorrhagic stroke was generally higher as the size of the residential area increased (*P* < 0.001) and was highest among the capital resident group (18.03%). The percentage of patients with relatively severe disabilities (NDR grade 3 or less) was the highest for metropolitan residents (9.54%).

Similar results were shown when comparing groups based on the presence or otherwise of UH. For patients residing in AD without UH, the average age was significantly high (*P* < 0.001), the proportion of men was significantly low (*P* = 0.002), the incidence of hypertension, diabetes, dyslipidemia, and CKD was significantly low (*P* = 0.014, *P* < 0.001, *P* < 0.001, and *P* < 0.001, respectively), and the NHIP level was significantly low (*P* < 0.001). In addition, the percentage of patients with ischemic stroke was significantly high (*P* = 0.003).

### Chronic phase long-term survival analysis

**Figure 2:**
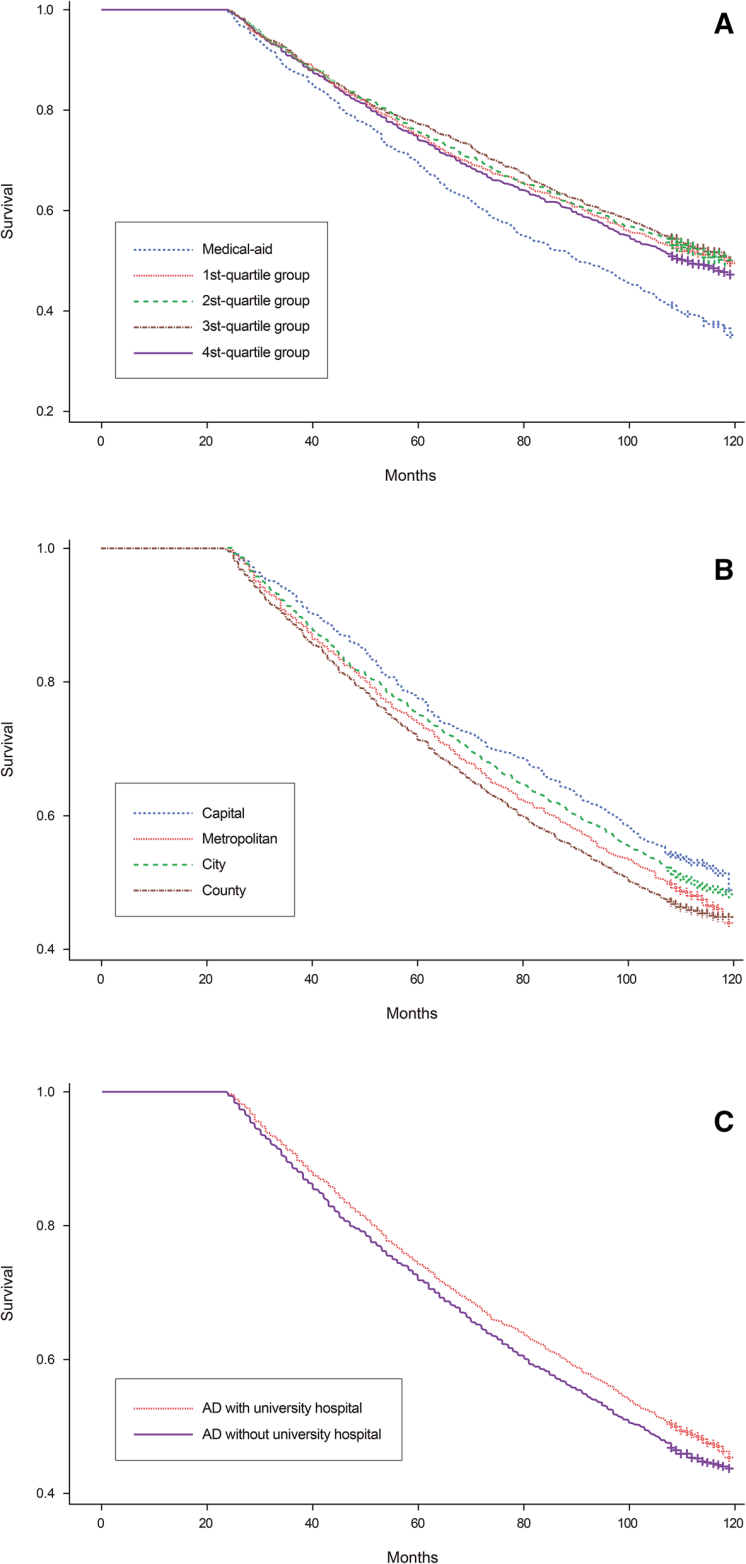
Kaplan–Meier survival curves according to: (**A**) each national health insurance premium (NHIP) level, (**B**) each residential area level, and (**C**) the presence or otherwise of a university hospital(s). The higher the NHIP level, the higher the survival rate (*P* < 0.001). The survival rate in the capital was the highest, and the county level showed the lowest survival rate (*P* < 0.001). Administrative districts with a university hospital(s) showed a higher survival rate compared to those without (*P* = 0.006).

Two- to ten-year cumulative survival tended to be higher at a high NHIP level (*P* < 0.001). However, while the 3rd quartile group showed the highest survival rate, the 4th quartile group showed the second-lowest survival rate, second only to the medical-aid group (Fig. 2A). In terms of residential areas, in the Model 1 analysis, the county level showed the lowest survival rate, while the capital showed the highest survival rate (*P* < 0.001) (Fig. 2B). In the Model 2 analysis, AD with UH showed a higher survival rate than AD with no UH (*P* = 0.006) (Fig. 2C).

Multivariable analysis using Cox proportional hazards models revealed that the NHIS-covered groups (1st–4th quartile groups) showed significantly lower chronic mortality risk than the medical-aid group. In contrast to univariate analysis results, the 4th quartile group showed the lowest chronic phase mortality risk (adjusted hazards ratio [HR] 0.71; 95% confidence interval [CI] 0.65–0.79; *P* < 0.001), followed by the 1st quartile group (adjusted HR 0.77; 95% CI 0.68–0.88; *P* < 0.001). These findings were consistent in the Cox proportional model without regional factors (Supplementary Table 6). Compared to capital residents, metropolitan residents had a significantly higher risk of chronic phase mortality (adjusted HR 1.15; 95% CI 1.03–1.29; *P* = 0.015). The mortality risk of city and county residents was not significantly higher compared to that of capital residents. In the Cox proportional model without NHIP levels, county residents showed significantly higher chronic phase mortality risk than capital residents (adjusted HR 1.15; 95% CI 1.03–1.29; *P* = 0.014) (Supplementary Table 7). In the Model 2 analysis, there was no significant difference in the risk of chronic phase mortality between the two groups (*P* = 0.218) (Table 3).

**Table 3:** Risk factor analysis for chronic phase mortality after stroke using Cox proportional-hazards models.

Variable	Model 1			Model 2		
	Adjusted HR	95% CI	*P*-value	Adjusted HR	95% CI	*P*-value
**NHIP levels**						
Medical-aid	1.00			1.00		
1st quartile	0.77	0.68–0.88	< 0.001	0.77	0.68–0.87	< 0.001
2nd quartile	0.79	0.70–0.90	< 0.001	0.79	0.70–0.89	< 0.001
3rd quartile	0.79	0.70–0.88	< 0.001	0.78	0.70–0.88	< 0.001
4th quartile	0.71	0.65–0.79	< 0.001	0.71	0.64–0.79	< 0.001
**Residential areas**						
Capital	1.00			Not included		
Metropolitan	1.15	1.03–1.29	0.015	Not included		
City	1.06	0.96–1.17	0.287	Not included		
County	1.12	1.00–1.26	0.053	Not included		
AD with UH	Not included			1.00		
AD without UH	Not included			1.04	0.98–1.12	0.218
Male	1.50	1.40–1.60	< 0.001	1.50	1.40–1.60	< 0.001
**Age groups**						
40–49	1.00			1.00		
50–59	1.46	1.01–2.13	0.046	1.48	1.02–2.15	0.040
60–69	2.54	1.79–3.60	< 0.001	2.55	1.80–3.62	< 0.001
70–79	5.33	3.78–7.51	< 0.001	5.36	3.81–7.55	< 0.001
≥ 80	11.77	8.34–16.59	< 0.001	11.82	8.38–16.66	< 0.001
**Stroke subtypes**						
SAH	1.00			1.00		
ICH	1.69	1.28–2.25	< 0.001	1.69	1.27–2.24	< 0.001
Cerebral infarction	1.55	1.19–2.03	0.001	1.55	1.19–2.04	0.001
Unspecified	1.47	1.06–2.04	0.021	1.45	1.05–2.02	0.025
Hypertension	0.95	0.87–1.03	0.190	0.94	0.87–1.03	0.167
Diabetes	1.15	1.07–1.23	< 0.001	1.15	1.07–1.23	< 0.001
Dyslipidemia	0.77	0.72–0.83	< 0.001	0.77	0.72–0.83	< 0.001
CAD	1.01	0.91–1.13	0.819	1.01	0.91–1.13	0.818
AF	1.39	1.24–1.55	< 0.001	1.39	1.24–1.56	< 0.001
CKD	2.06	1.73–2.45	< 0.001	2.07	1.73–2.46	< 0.001
**Disability grades**						
Ungraded	1.00			1.00		
Grade 6	0.62	0.34–1.16	0.138	0.63	0.34–1.17	0.145
Grade 5	0.67	0.40–1.14	0.139	0.68	0.40–1.15	0.146
Grade 4	1.39	1.10–1.77	0.007	1.38	1.09–1.76	0.008
Grade 3	1.13	0.89–1.44	0.308	1.15	0.90–1.45	0.262
Grade 2	1.52	1.24–1.86	< 0.001	1.52	1.24–1.87	< 0.001
Grade 1	2.18	1.88–2.52	< 0.001	2.19	1.89–2.53	< 0.001

*HR* Hazard ratio, *CI* confidence interval, *NHIP* national health insurance premium, *AD* administrative districts, *UH* university hospital, *SAH* subarachnoid hemorrhage, *ICH* intracranial hemorrhage, *CAD* coronary artery disease, *AF* atrial flutter/fibrillation, *CKD* chronic kidney disease.

In the Model 1 Cox regression analysis, the risk of chronic phase mortality was higher in the older age group. In addition, men had a significantly higher risk of death than women (adjusted HR 1.50; 95% CI 1.40–1.60; *P* < 0.001). Among the comorbidities, the presence of diabetes (adjusted HR 1.15; 95% CI 1.07–1.23; *P* < 0.001), AF (adjusted HR 1.39; 95% CI 1.24–1.55; *P* < 0.001), and CKD (adjusted HR 2.06; 95% CI 1.73–2.45; *P* < 0.001) significantly increased the risk of chronic phase mortality, whereas having dyslipidemia (adjusted HR 0.77; 95% CI 0.72–0.83; *P* < 0.001) significantly lowered the risk. The risk of mortality was significantly higher in other stroke subtypes than in the subarachnoid hemorrhage group. According to NDR grades, grade 1 (adjusted HR 2.18; 95% CI 1.88–2.52; *P* < 0.001), grade 2 (adjusted HR 1.52; 95% CI 1.24–1.86; *P* < 0.001), and grade 4 (adjusted HR 1.39; 95% CI 1.10–1.77; *P* = 0.007) showed a significantly higher risk of chronic phase mortality than the ungraded group (Table 3).

## Discussion

This study investigated the effects of NHIP level and residential area on chronic phase long-term survival after stroke. This study contributes to the field in that it provides an analysis that reflects the socioeconomic status based on the Korea NHIS and the degree of medical accessibility according to geographic distributions in patients with stroke. Notably, this study curtailed inter-patient variability by excluding deaths within 24 months of stroke onset. We considered that effective population targeting was important when researching stroke outcomes, as Cramer et al.^18^ also contended. Thus, we limited our patients to those who survived at least two years after stroke for the chronic survival analysis, making our study unique. This study design also related to the temporal aspect of the functional recovery after the stroke. Functional recovery after stroke generally shows nonlinear and logarithmic patterns^19^. Further, the functional decline becomes apparent after two years from stroke onset^20^. Therefore, we excluded deaths in the first two years because we intended to perform a survival analysis in chronic stage survivors who had established disabilities and were assigned disability grades. Additionally, this distinction was derived from the unique circumstance in South Korea. After the stroke onset, the Korean NHIS universally covers the post-stroke rehabilitation treatment for two years. Consequently, by including and analyzing stroke survivors at the time of 24 months after the stroke onset, we could focus more on the effect of the defined variables on the chronic phase of stroke.

The principal finding of this study was that the lower the NHIP level, the higher the risk of death in the chronic phase after stroke. Meanwhile, it was not consistent that differences in residential areas were independent risk factors for chronic phase mortality after stroke in our Cox proportional hazard models. These findings suggest that the NHIP level has a more significant effect on the risk of chronic death than geographic factors. There are various possible reasons for the high chronic phase mortality after stroke in the lower NHIP group. We believed that one reason for the higher mortality in the medical-aid group was the higher proportion of those aged 80 years or older in this group than other groups even when patient age was adjusted for in the multivariable analysis. In addition, even though our nested Cox-proportional models did not show the significant interaction between two primary contributing factors, we found that the smaller the residential area, the higher the percentage of patients in the medical-aid group tended to be.

The percentage of comorbidities tended to be greater in higher NHIP levels and larger residential areas. This finding indicates that the lower the socioeconomic status and the smaller the residential area, the more limited accessibility is likely to medical institutions following symptom awareness and disease events^21^. Limited accessibility to medical institutions means that the quality of care for stroke management is lowered during the acute and chronic phases of stroke^22^. In Model 1, with capital residents as the reference group, the risk of chronic phase mortality was significantly higher in metropolitan areas than in city or county areas. This may be because the percentage of patients with a relatively severe disability was highest among metropolitan residents. The model without NHIP levels supported these findings with significantly higher mortality risk in the county area, suggesting that smaller residential areas could be an independent risk factor for the chronic phase mortality in stroke.

This study’s findings support the need to address issues concerning residential areas as well as NHIP levels affecting the stroke care system and healthcare resources to ensure more effective management of patients with stroke. In practice, efforts have been made in South Korea to prevent the inefficient concentration of patients within tertiary medical institutions and expand high-quality stroke management centers in various regions. Regional cardio-cerebrovascular disease centers have been established across South Korea. Such centers have been assessed on their adequacy in cardio-cerebrovascular disease management, including stroke treatment^23^. In addition, the government is currently preparing to designate community-based cardio-cerebrovascular disease centers as sub-institutions at each regional center. By establishing these newly planned community centers, the government is striving to resolve issues regarding the current unequal concentration of emergency and specialized treatment systems for cardio-cerebrovascular diseases in certain regions^17^. At the same time, the Korean Ministry of Health and Welfare is spearheading a policy to create specialty hospitals to prevent the concentration of patients in large hospitals and help ensure the competitiveness and effectiveness of small and medium hospitals through specialization^24^. Hospitals can become specialty hospitals if they meet all the Health Insurance Review and Assessment services, including patient composition, facilities, and human resources^25^. Among specialty hospitals, CVD specialty hospitals are required to provide timely medical services by qualified personnel for acute reperfusion therapy and specialized and integrated management for stroke. Four institutions are currently designated as 4th batch CVD specialty hospitals in South Korea, specifically in Seoul (capital), Daegu (metropolitan), Cheongju (city), and Pohang (city)^26^. In addition, the Korea Stroke Society currently runs the primary stroke center certification system that certifies units that comply with established guidelines, which involves multidisciplinary staff and facilities specialized for stroke treatment^27,28^. These efforts are primarily intended to enhance hyperacute care of patients with stroke, improve patients’ long-term prognosis through early comprehensive rehabilitation, and provide additional education programs for maintenance and secondary prevention. Therefore, these efforts are directly related to the acute phase survival rate^13,29^ and can reduce chronic phase mortality and improve the long-term quality of life after stroke^30^. Model 1 showed no significant increase in the chronic phase mortality risk at the city or county level. In Model 2, even in AD without UH, no significant increase was observed in the chronic phase mortality risk. This finding suggests that the long-running efforts of the government and the Korea Stroke Society have been effective to some extent. However, further efforts are needed to narrow the inter-regional and socioeconomic gap in stroke management, which requires continuous and sufficient support for qualified regional medical institutions.

Our results showed that intracranial hemorrhage, ischemic stroke, and unspecified subtypes had a higher risk of chronic phase mortality than subarachnoid hemorrhage. This finding is contrary to a previous study, which found that hemorrhagic stroke had a high mortality risk^26,31,32^. However, our finding may be because the number of patients in the subarachnoid hemorrhage group (the reference group) was small, and many patients with low subarachnoid hemorrhage severity were enrolled since the study was conducted on patients who survived at least two years after stroke onset. Additionally, NDR grades 1 and 2 were identified as independent risk factors for chronic phase mortality, which accords with the results of previous studies reporting that severe disability in the chronic phase increased the risk of mortality^33^. There have been several studies on long-term survival after stroke. Sennfält et al.^31^ conducted a five-year survival analysis of 22,919 patients registered in the Swedish Stroke Register. The study identified age, male (gender), diabetes, independence, level of consciousness at admission, and loss to follow-up as independent risk factors. Boysen et al.^5^ conducted a 30-year survival study of 2,501 patients with first-ever stroke. Their study showed that age and hemorrhagic stroke were associated with low survival rates and that long-term survival gradually improved over time. In our study, older age, being a male, diabetes, AF, CKD, and severe disability were identified as independent risk factors for mortality in the chronic phase. Although our study included only chronic stage survivors, these results were comparable to previous findings^6,34–36^.

Our study is distinctive in finding that the presence of dyslipidemia lowered the risk of death after stroke. This may be due to the primary and secondary prevention effects of statin use on major vascular diseases among high-risk patients, as several studies have reported^37–39^. In other words, the use of statins among patients with dyslipidemia in our target patient group may have led to a prophylactic effect against cardiovascular events or recurrent stroke, reducing the risk of mortality in the chronic phase^40^. According to previous studies, the 10-year cumulative risk of recurrent stroke after a first-ever stroke event is 12–40%^41,42^. The 10-year cumulative risk of a cardiovascular event that occurs after stroke is also 5–39%^42,43^. Therefore, it could be considered that these two types of events have a significant effect on the prognosis and death of patients in the chronic phase of stroke. However, this study’s sample should be further studied to draw definite conclusions concerning the effects of statins on recurrent stroke or cardiovascular events.

This study had the following limitations. First, regarding ischemic stroke, which accounted for most of our patient group, a paradigm shift with reperfusion therapy occurred after 2015 with more aggressive administrating of intra-arterial mechanical thrombectomy^44^. Our study could not reflect this development, as it involved patients who experienced a stroke event in 2009. In situations where the newly developed reperfusion treatment guidelines are followed^45,46^, it may be the case that the effect of geographic factors on the chronic prognosis of patients with stroke is greater than that found in this study. Second, while the NHIP levels reflect each household’s economic level, they do not directly reflect the individual patient's economic level. If the two groups with insured and dependent persons under the Korean NHIS are separately analyzed, our findings can be further supported. Third, we used disability grades registered in the NDR system to indicate stroke severity. This usage had the advantage of reflecting the patient's disability status objectively at 24 months after onset, at which time we started the survival analysis. However, there may be patients who are not enrolled in the NDR system even though they have disabilities, particularly patients in the medical-aid group. We also considered that fewer patients registered as having NDR grades 5 and 6 might be related to minor disabilities not registered in the NDR system. Fourth, the dataset in this study was not sufficient to accurately present the degree of secondary stroke prevention and health care in the chronic phase. We could not specifically report secondary prevention care service variation by NHIP levels and regional differences in the same context. Lastly, we analyzed all-cause mortality as the end-point and could not specify the cause of death. The limitations mentioned above require further research through the combination of NHI data and individual clinical data.

The strengths of this study were as follows. Our study was based on an extensive, nationwide database. Through this, it was possible to analyze chronic phase mortality risk using various confounding factors in patients with stroke. The primary information such as NHIP level and residential area were objective and accurate based on the NHIS of South Korea. By presenting a model through multifaceted analysis, we could definitely confirm the effect of socioeconomic status and geographic factors on chronic phase mortality after stroke.

## Conclusions

Our results highlight the need for nationwide efforts to reduce inter-regional and socioeconomic discrepancies affecting stroke management in South Korea. For this, qualified hospitals such as cardio-cerebrovascular disease centers, CVD specialty hospitals, and primary stroke centers have been designated, which have a system for long-term management such as patient education, rehabilitation, secondary prevention, and acute stroke care. Further strengthening and supporting these systems can efficiently distribute medical resources for stroke management and improve long-term outcomes.

## Supplementary Information

Supplementary Tables.

## Data Availability

The data are not publicly available due to privacy and ethical restrictions of the Korean National Health Insurance data sharing system. The dataset used in this study can only be accessed by an authorized researcher through its own internal-networking system.
