# Supplementary material for: The effects of socioeconomic and geographic factors on chronic phase long-term survival after stroke in South Korea
Source: Sci Rep. 2022 Mar 14;12:4327. doi: 10.1038/s41598-022-08025-2 (PMC8921252; doi:10.1038/s41598-022-08025-2)
Supplement: Supplementary file 1 — Supplementary Tables. [file 41598_2022_8025_MOESM1_ESM.pdf]

Supplementary Materials

The Effects of Socioeconomic and Geographic Factors on Chronic Phase Long-Term Survival after Stroke in South Korea

Dougho Park[1]†, Su Yun Lee[2]†, Eunhwan Jeong[2], Daeyoung Hong[3], Mun-Chul Kim[3], Jun Hwa Choi[4], Eun Kyong Shin[5], Kang Ju Son[6,7] *, Hyoung Seop Kim[8] *

[1]Department of Rehabilitation Medicine, Brain and Vascular Center, Pohang Stroke and Spine Hospital, Pohang, Republic of Korea

[2]Department of Neurology, Brain and Vascular Center, Pohang Stroke and Spine Hospital, Pohang, Republic of Korea

[3]Department of Neurosurgery, Brain and Vascular Center, Pohang Stroke and Spine Hospital, Pohang, Republic of Korea

[4]Department of Quality Improvement, Pohang Stroke and Spine Hospital, Pohang, Republic of Korea

[5]Department of Sociology, Korea University, Seoul, Republic of Korea

[6]Department of Research and Analysis Team, National Health Insurance Service Ilsan Hospital, Goyang, Republic of Korea

[7]Department of Biostatistics and Computing, Yonsei University Graduate School, Seoul, Republic of Korea

[8]Department of Physical Medicine and Rehabilitation, National Health Insurance Service Ilsan Hospital, Goyang, Republic of Korea

Supplementary Table 1. Administrative districts where at least one or more university hospitals are located in South Korea. (in 2018)

	Name of City/County
Capital	Seoul
Metropolitan	Busan, Incheon, Daegu, Daejeon, Gwangju, Ulsan,
Gyeonggi	Goyang, Seongnam, Anyang, Bucheon, Uijeongbu, Hwaseong, Suwon, Guri, Gunpo, Ansan, Yongin
Chungcheongnam	Cheonan
Chungcheongbuk	Chungju, Cheongju
Gangwon	Chuncheon, Wonju, Gangneung
Jeollabuk	Jeonju, Iksan
Jeollanam	Hwasun
Gyeongsangbuk	Gyeongju, Gumi
Gyeongsangnam	Changwon, Jinju, Yangsan
Jeju Island	Jeju

Supplementary Table 2. ICD-10 codes for defined comorbidities

Disease	ICD-10 codes
Hypertension	I10, I11, I12, I13, I15
Diabetes	E10, E11, E12, E13, E14
Dyslipidemia	E78
CAD	I25
AF	I48
CKD	N18

ICD, International Classification of Disease; CAD, coronary artery disease; AF, atrial flutter/fibrillation; CKD, chronic kidney disease.

Supplementary Table 3. Modified Barthel index according to the national disability registration grading system.

Grade	Modified Barthel index
1	≤32
2	33–53
3	54–69
4	70–80
5	81–89
6	90–96
Ungraded	≥97

Supplementary Table 4. Monthly national health insurance premium amount by each premium quantile (in 2018)

Level	Insurance premium quantile	Korean Won
Group 1	Medical-aid	0
Group 2	1	3,660
	2	9,890
	3	40,560
	4	46,800
	5	49,920
Group 3	6	53,040
	7	56,160
	8	62,400
	9	68,640
	10	74,880
Group 4	11	78,000
	12	84,240
	13	93,600
	14	109,200
	15	124,800
Group 5	16	140,400
	17	156,000
	18	187,200
	19	218,400
	20	312,000

*1$ = 1,150 Korean Won

Supplementary Table 5. Population size by each residential area level (in 2018)

	Residential Area	Population
Group 1	Capital	9,765,623
Group 2	Metropolitan, median (range)	1,975,853 (1,155,623−3,441,453)
Group 3	City, median (range)	232,327 (43,731−1,201,166)
Group 4	County, median (range)	44,633 (9,832−118,828)

Supplementary table 6. Cox proportional-hazards models without geographic factors

Variable		Adjusted HR	95% CI	*P*-value
NHIP levels				
	Medical-aid	1.00		
	1st quartile	0.77	0.68–0.87	<0.001
	2nd quartile	0.79	0.70–0.89	<0.001
	3rd quartile	0.78	0.69–0.87	<0.001
	4th quartile	0.71	0.64–0.78	<0.001
Male		1.50	1.40–1.60	<0.001
Age groups				
	40–49	1.00		
	50–59	1.48	1.02–2.14	0.041
	60–69	2.55	1.80–3.62	<0.001
	70–79	5.38	3.82–7.57	<0.001
	≥ 80	11.84	8.39–16.69	<0.001
Stroke subtypes				
	SAH	1.00		
	ICH	1.69	1.27–2.25	<0.001
	Cerebral infarction	1.56	1.19–2.04	0.001
	Unspecified	1.46	1.05–2.03	0.023
Hypertension		0.94	0.87–1.02	0.159
Diabetes		1.15	1.07–1.23	<0.001
Dyslipidemia		0.77	0.72–0.83	<0.001
CAD		1.01	0.91–1.13	0.823
AF		1.39	1.24–1.56	<0.001
CKD		2.06	1.73–2.45	<0.001
Disability grades				
	Ungraded	1.00		
	Grade 6	0.63	0.34–1.17	0.143
	Grade 5	0.68	0.40–1.14	0.145
	Grade 4	1.39	1.09–1.76	0.008
	Grade 3	1.15	0.90–1.45	0.265
	Grade 2	1.52	1.24–1.86	<0.001
	Grade 1	2.18	1.89–2.52	<0.001

HR, Hazard ratio; CI, confidence interval; NHIP, national health insurance premium; SAH, subarachnoid hemorrhage; ICH, intracranial hemorrhage; CAD, coronary artery disease; AF, atrial flutter/fibrillation; CKD, chronic kidney disease.

Supplementary table 7. Cox proportional-hazards models without NHIP level

Variable	Model 1			Model 2		
	Adjusted HR	95% CI	*P*-value	Adjusted HR	95% CI	*P*-value
Residential areas						
Capital	1.00					not included
Metropolitan	1.17	1.04–1.31	0.007			not included
City	1.07	0.97–1.18	0.176			not included
County	1.15	1.03–1.29	0.014			not included
AD with UH			not included	1.00		
AD without UH			not included	1.06	0.99–1.13	0.107
Male	1.47	1.37–1.57	<0.001	1.47	1.37–1.57	<0.001
Age groups						
40–49	1.00			1.00		
50–59	1.43	0.99–2.08	0.059	1.45	1.00–2.10	0.052
60–69	2.45	1.73–3.46	<0.001	2.46	1.74–3.49	<0.001
70–79	5.08	3.61–7.15	<0.001	5.12	3.63–7.19	<0.001
≥ 80	11.31	8.02–15.94	<0.001	11.36	8.06–16.01	<0.001
Stroke subtypes						
SAH	1.00			1.00		
ICH	1.71	1.29–2.27	<0.001	1.70	1.28–2.26	<0.001
Cerebral infarction	1.58	1.20–2.06	<0.001	1.58	1.21–2.07	<0.001
Unspecified	1.50	1.08–2.08	0.016	1.07	1.07–2.05	0.019
Hypertension	0.95	0.87–1.03	0.198	0.94	0.87–1.03	0.169
Diabetes	1.14	1.07–1.23	<0.001	1.14	1.07–1.23	<0.001
Dyslipidemia	0.76	0.71–0.82	<0.001	0.76	0.71–0.81	<0.001
CAD	1.01	0.91–1.13	0.817	1.01	0.91–1.13	0.821
AF	1.36	1.22–1.52	<0.001	1.37	1.22–1.53	<0.001
CKD	2.06	1.73–2.45	<0.001	2.07	1.73–2.46	<0.001
Disability grades						
Ungraded	1.00			1.00		
Grade 6	0.61	0.33–1.14	0.119	0.61	0.33–1.14	0.125
Grade 5	0.67	0.40–1.13	0.135	0.68	0.40–1.14	0.145
Grade 4	1.36	1.07–1.73	0.012	1.35	1.06–1.71	0.015
Grade 3	1.12	0.88–1.42	0.360	1.13	0.89–1.44	0.307
Grade 2	1.49	1.21–1.82	<0.001	1.49	1.22–1.83	<0.001
Grade 1	2.13	1.85–2.47	<0.001	2.14	1.85–2.48	<0.001

NHIP, national health insurance premium; HR, Hazard ratio; CI, confidence interval; AD, administrative districts; UH, university hospital; SAH, subarachnoid hemorrhage; ICH, intracranial hemorrhage; CAD, coronary artery disease; AF, atrial flutter/fibrillation; CKD, chronic kidney disease.
